# Artificial Intelligence: A Clarification of Misconceptions, Myths and Desired Status

**DOI:** 10.3389/frai.2020.524339

**Published:** 2020-12-23

**Authors:** Frank Emmert-Streib, Olli Yli-Harja, Matthias Dehmer

**Affiliations:** ^1^ Predictive Society and Data Analytics Lab, Faculty of Information Technology and Communication Sciences, Tampere University, Tampere, Finland; ^2^ Institute of Biosciences and Medical Technology, Tampere, Finland; ^3^ Computational System Biology, Faculty of Medicine and Health Technology, Tampere University, Finland; ^4^ Institute for Systems Biology, Seattle, WA, United States; ^5^ Department of Mechatronics and Biomedical Computer Science, UMIT, Hall in Tyrol, IL, Austria; ^6^ Department of Computer Science, Swiss Distance University of Applied Sciences, Brig, Switzerland; ^7^ College of Artificial Intelligence, Nankai University, Tianjin, China

**Keywords:** artificial intelligence, artificial general intelligence, machine learning, statistics, data science, deep neural networks, data mining, pattern recognition

## Abstract

The field artificial intelligence (AI) was founded over 65 years ago. Starting with great hopes and ambitious goals the field progressed through various stages of popularity and has recently undergone a revival through the introduction of deep neural networks. Some problems of AI are that, so far, neither the “intelligence” nor the goals of AI are formally defined causing confusion when comparing AI to other fields. In this paper, we present a perspective on the desired and current status of AI in relation to machine learning and statistics and clarify common misconceptions and myths. Our discussion is intended to lift the veil of vagueness surrounding AI to reveal its true countenance.

## 1. Introduction

Artificial intelligence (AI) has a long tradition going back many decades. The name *artificial intelligence* was coined by McCarthy at the Dartmouth conference in 1956 that started a concerted endeavor of this research field which continues to date (McCarthy et al., 2006). The initial focus of AI was on *symbolic models and reasoning* followed by the first wave of *neural networks* (NN) and *expert systems* (ES) (Rosenblatt, 1957; Newel and Simon, 1976; Crevier, 1993). The field experienced a severe setback when Minsky and Papert demonstrated problems with perceptrons in learning non-linear separable functions, e.g., the exclusive OR (XOR) (Minsky and Papert, 1969). This significantly affected the progression of AI in the following years especially in neural networks . However, in the 1980s neural networks made a comeback through invention of the *back-propagation algorithm* (Rumelhart et al., 1986). Later in the 1990s research about *intelligent agents* garnered broad interest (Wooldridge and Jennings, 1995) exploring for instance coupled effects of perceptions and actions (Wolpert and Kawato, 1998; Emmert-Streib, 2003). Finally, in the early 2000s big data became available and led to another revival of neural networks in the form of deep neural networks (DNN) (Hochreiter and Schmidhuber, 1997; Hinton et al., 2006; O’Leary, 2013; LeCun et al., 2015).

During these years, AI has achieved great success in many different fields including robotics, speech recognition, facial recognition, healthcare, and finance (Bahrammirzaee, 2010; Brooks, 1991; Krizhevsky et al., 2012; Hochreiter and Schmidhuber, 1997; Thrun, 2002; Yu et al., 2018). Importantly, these problems do not all fall within one field, e.g., computer science, but span a multitude of disciplines including psychology, neuroscience, economy, and medicine. Given the breath of AI applications and the variety of different methods used, it is no surprise that seemingly simple questions, e.g., regarding the aims and goals of AI are obscured especially for those scientists who did follow the field since its inception. For this reason, in this paper, we discuss the desired and current status of AI regarding its definition and provide a clarification for the discrepancy. Specifically, we provide a perspective on AI in relation to machine learning and statistics and clarify common misconceptions and myths.

Our paper is organized as follows. In the next section, we discuss the desired and current status of artificial intelligence including the definition of “intelligence” and strong AI. Then we clarify frequently encountered misconceptions about AI. Finally, we discuss characteristics of methods from artificial intelligence in relation to machine learning and statistics. The paper is completed with concluding remarks.

## 2. What Is Artificial Intelligence?

We begin our discussion by clarifying the meaning of artificial intelligence itself. We start by presenting attempts to define “intelligence” followed by informal characterizations of AI as the former problem is currently unresolvable.

### 2.1. Defining “Intelligence” in Artificial Intelligence

From the name “artificial intelligence” it seems obvious that AI dealswith an artificial–not natural - form of intelligence. Hence, defining ‘intelligence’ in a precise way will tell us what AI is about. Unfortunately, currently, there is no such definition available that would be generally accepted by the community. For an extensive discussion of the difficulties encountered when attempting to provide such a definition see, e.g., (Legg and Hutter, 2007; Wang, 2019).

Despite the lack of such a generally accepted definition, there are various attempts. For instance, a recent formal measure has been suggested by (Legg and Hutter, 2007). Interestingly, the authors start from several informal definitions of *human* intelligence to define machine intelligence formally. The resulting measure is given by

$$\text{universal intelligence of agent }\pi =ϒ\left(\pi \right)=\underset{\mu \in E}{\sum }2^{-K\left(\mu \right)}V_{\mu }^{\pi }.$$ (1)

Here *π* is an agent, *K* the Kolmogorov complexity function, *E* the set of all environments, *µ* one particular environment, $2^{-K\left(\mu \right)}$ the algorithmic probability distribution over an environment and $V_{\mu }^{\pi }$ a value function. Overall, ϒ(π) is called the universal intelligence of agent *π* (Legg and Hutter, 2007). Informally, Eq 1 gives a measure for intelligence as the ability of an agent to achieve goals in a wide range of environments (Legg and Hutter, 2007).

A general problem with the definition given in Eq 1 is that its form is rather cumbersome and unintuitive, and its exact practical evaluation is not possible because the Kolmogorov complexity function K is not computable but requires an approximation. A further problem is to perform intelligence tests because, e.g., a Turing test (Turing, 1950) is insufficient (Legg and Hutter, 2007), for instance, because an agent could appear intelligent without actually being intelligent (Block, 1981).

A good summary of the problem in defining “intelligence” and AI is given in (Winston and Brown, 1984), who state that “Defining intelligence usually takes a semester-long struggle, and even after that I am not sure we ever get a definition really nailed down. But operationally speaking, we want to make machines smart.” In summary, there is currently neither a generally accepted definition of “intelligence” nor tests that could be used to identify “intelligence” reliably.

In spite of this lack of a general definition of “intelligence,” there is a philosophical separation of AI systems based on this notion. The so called weak AI hypothesis states that “machines could act as if they were intelligent” whereas the strong AI hypothesis asserts “that machines that do so are actually thinking (not just simulating thinking)” (Russell and Norvig, 2016). The latter in particular is very controversial and an argument against a strong AI is the Chinese room (Searle, 2008). We would like to note that strong AI hasrecently been rebranded as *artificial general intelligence* (AGI) (Goertzel and Pennachin, 2007; Yampolskiy and Fox, 2012).

### 2.2. Informal Characterizations of Artificial Intelligence

Since there is no generally accepted definition of “intelligence” AI has been characterized informally from its beginnings. For instance, in (Winston and Brown, 1984) it is stated that “The primary goal of Artificial Intelligence is to make machines smarter. The secondary goals of Artificial Intelligence are to understand what intelligence is (the Nobel laureate purpose) and to make machines more useful (the entrepreneurial purpose)”. Kurzweil noted that artificial intelligence is “The art of creating machines that perform functions that require intelligence when performed by people” (Kurzweil et al., 1990). Furthermore, Feigenbaum (Feigenbaum, 1963) said “artificial intelligence research is concerned with constructing machines (usually programs for general-purpose computers) which exhibit behavior such that, if it were observed in human activity, we would deign to label the behavior ‘intelligent’.” The latter reminds one of a Turing test of intelligence and that a measure for intelligence is connected to such a test; see our discussion in the last section.

Feigenbaum further specifies that “One group of researchers is concerned with simulating human information-processing activity, with the quest for precise psychological theories of human cognitive activity” and “A second group of researchers is concerned with evoking intelligent behavior from machines whether or not the information processes employed have anything to do with plausible human cognitive mechanisms” (Feigenbaum, 1963). Similar distinctions have been made in (Simon, 1969; Pomerol, 1997). Interestingly, the first point addresses a natural–not artificial–form of cognition showing that some scientists even cross the boundary from artificial to biological phenomena.

From this follows, that from its beginnings, AI had high aspirations focusing on ultimate goals centered around *intelligent* and *smart* behavior rather than on simple questions as represented, e.g., by classification or regression problems as discussed in statistics or machine learning. This also means that AI is not explicitly data-focused but assumes the availability of data which would allow the studying of such high-hanging questions. This relates also, e.g., to probabilistic or symbolic approaches (Koenig and Simmons, 1994; Hoehndorf and Queralt-Rosinach, 2017). Importantly, this is in contrast to data science which places data at the center of the investigation and develops estimation techniques for extracting the optimum of information contained in data set(s) possibly by applying more than one method (Emmert-Streib and Dehmer, 2019a).

### 2.3. Current Status

From the above discussion, it seems fair to assert that we neither have a generally accepted, formal (mathematical) definition of “intelligence” nor do we have one succinct informal definition of AI that would go beyond its obvious meaning. Instead, there are many different characterizations and opinions about what AI should be (Wang, 2006).

Given this deficiency it is not surprising that there are many misconceptions and misunderstandings about AI in general. In the following section, we discuss some of these.

## 3. Common Misconceptions and Myths

In this section, we discuss some frequently encountered misconceptions about AI and clarify some false assumptions.

*AI aims to explain how the brain works*. No, because brains occur only in living (biological) beings and not in artificial machines. Instead, fields studying the molecular biological mechanisms of natural brains are neuroscience and neurobiology. Whether AI research can contribute to this question in some way is unclear but so far no breakthrough contribution has been made. Nevertheless, it is unquestionable that AI research wasinspired by neurobiology from its very beginnings (Fan et al., 2020) and one prominent example for this is Hebbian learning (Hebb, 1949) or extensions thereof (Emmert-Streib, 2006).

AI methods work similar *to the brain*. No, this is not true; even if the most popular methods of AI are called neural networks which are inspired by biological brains. Importantly, despite the name “neural network” such models do not present physiological neural models because neither the model of a neuron nor the connectivity between the neurons in neural networks is biologically plausible nor realistic. That means neither the connectivity structure of convolutional neural networks nor that of deep feedforward neural networks or other deep learning architectures are biologically realistic. In contrast, a physiological model of a biological neuron is the Hodgkin-Huxley model (Hodgkin and Huxley, 1952) or the FitzHugh-Nagumo model (Nagumo et al., 1962) and the large-scale connectivity of the brain is to date largely unknown.

*Methods from AI have a different purpose as methods from machine learning or statistics*. No, the general purpose of all methods from these fields is to analyze data. However, each field introduced different methods with different underlying philosophies. Specifically, the philosophy of AI is to aim at ultimate goals, which are possibly unrealistic, rather than to answer simple questions. As a note, we would like to remark that any manipulation of data stored in a computer, is a form of data analysis. Interestingly, this is even true for agent-based systems, e.g., robotics, which incrementally gather data via the interaction with an environment. Kaplan and Haenlein phrased this nicely as “a system’s ability to correctly interpret external data, to learn from such data, and to use those learnings to achieve specific goals and tasks through flexible adaptation”, when defining AI (Kaplan and Haenlein, 2019).

AI is a technology. No, AI is a methodology. That means the methods behind AI are (mathematical) learning algorithms that adjust the parameters of methods via learning rules. However, when implementing AI methods certain problems may require an optimization of the method in combination with computer hardware, e.g., by using a GPU, in order to improve the computation time it takes to execute a task. The latter combination may give the impression that AI is a technology but by downscaling a problem one can always reduce the hardware requirements, demonstrating the principle workings of a method, potentially for toy examples. Importantly, in the above argument we emphasized the intellectual component of AI. It is clear that AI cannot be done with a pencil and piece of paper, hence, a computer is always required and a computer is a form of technology. However, the intellectual component of AI is not the computer itself but the software implementing learning rules.

AI makes computers think. From a scientific point of view, no, because similar to the problems of defining “intelligence” there is currently no definition of “thinking”. Also thinking is in general associated with humans who are biological beings rather than artificial machines. In general, this point is related to the goals of strong AI and the counter argument by Searle (Searle, 2008).

Why does AI appear more mythical than machine learning or statistics? Considering the fact that those fields serve a similar purpose (see above) this is indeed strange. However, we think that the reason therefore is twofold. First, the vague definition of AI leaves much room for guesswork and wishful thinking which can be populated by a wide range of philosophical considerations. Second, the high aspirations of AI enable speculations about ultimate or futuristic goals like “making machines think” or “making machines human-like”.

*Making machines behave like humans is optimal*. At first, this sounds reasonable but let us consider an example. Suppose there is a group of people and the task is to classify handwritten numbers. This is a difficult problem because the hand writing can be difficult to read. For this reason, one cannot expect that all people will achieve the optimal score, but some people perform better than others. Hence, the behavior of every human is not optimal compared to the maximal score or even the best performing human. Also, if we give the same group of people, a number of different tasks to solve, then it is unlikely that the same person will always perform best. Altogether, it does not make sense to make a computer behave like humans because most people do not perform optimally, regardless of what task we consider. So, what it actually means is to make a computer perform like the best performing human. For one task this may actually mimic the behavior of one human, however, for several tasks this will correspond to the behavior of a different human for every task. Hence, such a *super human* does not exist. That means if a machine can solve more than one task it does not make sense to compare it to one human because such a person does not exist. Hence, the goal is to make machines behave like an ideal super human.

*When will the ultimate goals of AI be reached?* Over the years there have been a number of predictions. For instance, Simon predicted in 1965 that “Machines will be capable, within twenty years, of doing any work a man can do” (Simon, 1965), Minsky stated in 1967 that “Within a generation … the problem of creating artificial intelligence will substantially be solved” (Minsky, 1967) and Kurzweil predicted in 2005 that strong AI, which he calls singularity, will be realized by 2045 (Kurzweil, 2005). Obviously, the former two predictions turned out to be wrong and the latter one remains in the future. However, predictions about undefined entities are vague (see our discussion about intelligence above) and cannot be evaluated systematically. Nevertheless, it is unquestionable that methods from AI make a continuous contribution to many areas in science and industry.

From the above discussion one realizes that metaphors are frequently used when talking about AI but those are not meant to be understood in a precise way but more as a motivation or stimulation. The origin of this might be related to the community behind AI which is considerably different from the more mathematics oriented communities in statistics or machine learning.

## 4. Discussion

**FIGURE 1:**
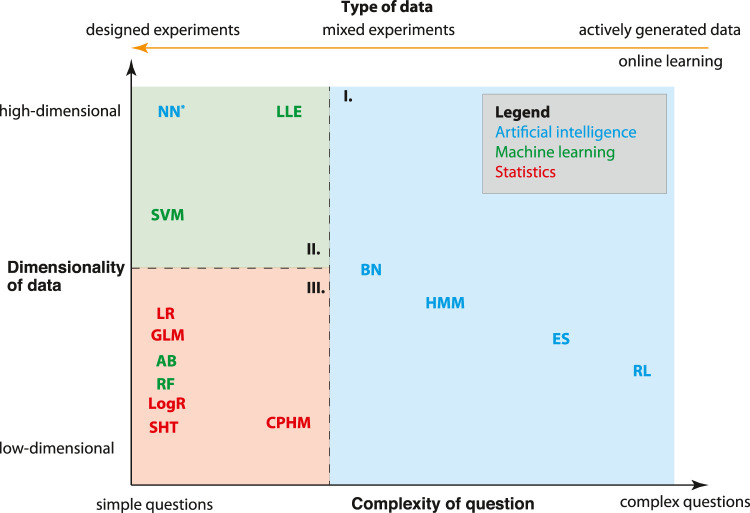
A simplified, graphical overview of properties of core (and base) methods from artificial intelligence (AI), machine learning (ML) and statistics. The *x*-axis indicates simple **(left)** and complex **(right)** questions a method can study whereas the *y*-axis indicates low- and high-dimensional methods. In addition, there is an orange axis **(top)** indicating different data-types. Overall, one can distinguish three regions where either methods from artificial intelligence (blue), machine learning (green) or statistics (red) dominate. See Table 1 for acronyms.

In the previous sections, we discussed various aspects of AI and their limitations. Now we aimfor a general overview of the relations between methods in artificial intelligence, machine learning and statistics. In Table 1 we show a list of core methods from artificial intelligence, machine learning and statistics. Here “core” refers to methods that can be considered as characteristic for a field, e.g., hypothesis testing for statistics, support vector machines for machine learning or neural networks for artificial intelligence. Each of these methods has attributes with respect the capabilities of the methods. In the following, we consider three such attributes as the most important; 1) the complexity of questions to be studied, 2) the dimensionality of data to be processed and 3) the type of data that can be analyzed. In Figure 1, we show a simplified, graphical overview of these properties (the acronyms are given in Table 1). We would like to highlight that these distinctions present our own, idealized perspective shared by many. However, alternative views and perspectives are possible.

**TABLE 1:** List of popular, core artificial intelligence, machine learning and statistics methods representing characteristic models of those fields.

Model	Application	Refs.
Neural networks (NN)	Function approximation, classification	Rosenblatt (1957), Schmidhuber (2015)
Expert system (ES)	Knowledge-based decisions	Hayes-Roth et al. (1983)
Hidden markov models (HMM)	Sequential symbol processing	Rabiner (1989)
Bayesian networks (BN)	Uncertain reasoning	Pearl (1988), Scutari (2010)
Reinforcement learning (RL)	Decision planing	Kaebeling et al. (1996), Sutton and Barto (1998)
Support vector machines (SVM)	Classification	Vapnik (1995), Schölkopf and Smola (2002)
Adaptive boosting (AB)	Classification	Freund and Schapire (1997)
XGBoost (XGB)	Classification	Chen and Guestrin (2016)
Locally linear embedding (LLE)	Nonlinear dimensionality reduction	Roweis and Saul (2000)
Random forests (RF)	Classification	Breiman (2001)
Linear regression (LR)	Regression	Weisberg (2005), Emmert-Streib and Dehmer (2019b)
Logistic regression (LogR)	Classification	Kleinbaum et al. (2002)
Generalized linear models (GLM)	Regression	Nelder and Wedderburn (1972), Dunn and Smyth (2018))
Statistical hypothesis testing (SHT)	Hypothesis testing	Sheskin (2004), Emmert-Streib and Dehmer (2019d)
Cox proportional hazard model (CPHM)	Survival analysis	Cox (1972), Kleinbaum and Klein (2005)

In general, there are many properties of methods one can use for a distinction. However, we start by focusing on only two such features. Specifically, the *x*-axis inFigure 1 indicates the question-type that can be addressed by a method from simple (left-hand side) to complex (right-hand side) questions, whereas the *y*-axis indicates the input dimensionality of the data from low-to high-dimensional. Here the dimensionality of the data corresponds to the length of a feature vector used as the input for an analysis method which is different to the number of samples which gives the total number of different feature vectors. Overall, inFigure 1 one can distinguish three regions where methods from artificial intelligence (blue), machine learning (green), or statistics (red) dominate. Interestingly, before the introduction of deep learning neural networks, region II. was entirely dominated by machine learning methods. For this reason we added a star to neural networks (NN) to indicate it as modern AI method. As one can see, methods from statistics are generally characterized by simple questions that can be studied in low-dimensional settings. Here by “simple” we do not mean “boring” or “uninteresting” but rather “specific” or “well defined”. Hence, from Figure 1 one can conclude that AI tends to address complex questions that do not fit well into a conventional framework, e.g., as represented by statistics. The only exception is neural networks.

For most of the methods shown in Table 1 exist extensions to the “base” method. For instance, a classical statistical hypothesis test is conducted just once. However, modern problems in genomics or the social sciences require the testing of thousands or millions of hypotheses. For this reason multiple testing corrections have been introduced (Farcomeni, 2008; Emmert-Streib and Dehmer, 2019c). Similar extensions can be found for most other methods, e.g., regression. However, when considering only the original core methods one obtains a simplified categorization for the domains of AI, ML and statistics, which can be summarized as follows:

• Traditional domain of artificial intelligence ⇒ Complex questions
• Traditional domain of machine learning ⇒ High-dimensional data
• Traditional domain of statistics ⇒ Simple questions

In Figure 1, we added one additional axis (orange) on top of the figure indicating different types of data. In contrast to the axes for the question-type and the dimensionality of input data, the scale of this axis is categorial, which means there is no smooth transition between the corresponding categories. Using this axis (feature) as an additional perspective, one can see that machine learning as well as statistics methods require data from designed experiments. This form of experiment corresponds to the conventional type of experiments in physics or biology, where the measurements follow a predefined plan called an experimental design. In contrast, AI methods frequently use actively generated data [also known as online learning (Hoi et al., 2018)] which become available in a sequential order. An example for this data type is the data a robot generates by exploring its environment or data generated by moves in games (Mnih et al., 2013).

We think it is important to emphasize that (neither) methods from AI (nor from machine learning or statistics) can be mathematically derived from a common, underlying methodological framework but they have been introduced separately and independently. In contrast, physical theories, e.g., about statistical mechanics or quantum mechanics, can be derived from a Hamiltonian formalism or alternatively from Fisher Information (Frieden and Frieden, 1998; Goldstein et al., 2013).

Maybe the most interesting insight from Figure 1 is that the current most successful AI methods, namely neural networks, do not address complex questions but simple ones (e.g., classification or regression) for high-dimensional data (Emmert-Streib et al., 2020). This is notable because it goes counter the tradition of AI taking on novel and complex problems. Also considering the current interest in futuristic problems, e.g., self-driving cars, automatic trading or health diagnostics this seems even more curious because it means such complex questions are addressed reductionistically dissecting the original problem into smaller subproblems rather than addressing them as a whole. Metaphorically, this may be considered as maturing process of AI settling after a rebellious adolescence against the limitations of existing fields like control theory, signal processing or statistics (Russell and Norvig, 2016). Whether it will remain this way, remains to be to be seen in the future.

Finally, if one considers also novel extensions for all base methods from AI, ML and statistics one can summarize the current state of these fields as follows:

Current domain of artificial intelligence, machine learning and statistics ⇒ Simple questions for high-dimensional data

This means that all fields seem to converge to simple question for high-dimensional data.

## 5. Conclusions

In this paper, we discussed the desired and current status of AI and clarified its goals. Furthermore, we put AI into perspective alongside machine learning and statistics and identified similarities and differences. The most important results can be summarized as follows:

(1) currently, no generally accepted definition of “intelligence” is available. ⇒ AI remains mathematically undefined, almost 65 years after its formal inception.
(2) the aspirations of AI are very high focusing on ambitious goals. ⇒ AI is not explicitly data focused–in contrast to data science.
(3) general AI methods do not provide neurobiological models of brain functions. ⇒ AI methods are merely means to analyze data - similar to methods from machine learning and statistics.
(4) addition: Also deep neural networks also do not provide neurobiological models of brain functions. ⇒ They are merely means to analyze data.
(5) the current most successful AI methods, i.e., deep neural networks, focus on simple questions (classification, regression) and high-dimensional data. ⇒ This goes counter traditional AI but is similar to contemporary machine learning and statistics.
(6) AI methods are not derived from a common mathematical formalism but have been introduced separately and independently. ⇒ There is no common conceptual framework that would unite the ideas behind different AI methods.

Finally, we would like to note that the closeness to applications of AI is certainly good for making the field practically relevant and for achieving an impact in the real world. Interestingly, this is very similar to a commercial product. A downside is that AI also comes with slogans and straplines used for marketing reasons just as those used for regular commercial products. We hope our article can help people look beyond the marketing definition of AI to see what the field is actually about from a scientific perspective.
